# Review of the Capacity to Accurately Detect the Temperature of Human Skin Tissue Using the Microwave Radiation Method

**DOI:** 10.3390/bios14050221

**Published:** 2024-04-28

**Authors:** Jingtao Wu, Jie Liu

**Affiliations:** 1 School of Information Science and Engineering, Southeast University, Nanjing 210096, China; jt_wu@seu.edu.cn; 2 The Faculty of Information Technology, Beijing University of Technology, Beijing 100124, China

**Keywords:** microwave radiometry, early diagnosis, precise measurement, non-contact, subcutaneous tissue temperature

## Abstract

Microwave radiometry (MWR) is instrumental in detecting thermal variations in skin tissue before anatomical changes occur, proving particularly beneficial in the early diagnosis of cancer and inflammation. This study concisely traces the evolution of microwave radiometers within the medical sector. By analyzing a plethora of pertinent studies and contrasting their strengths, weaknesses, and performance metrics, this research identifies the primary factors limiting temperature measurement accuracy. The review establishes the critical technologies necessary to overcome these limitations, examines the current state and prospective advancements of each technology, and proposes comprehensive implementation strategies. The discussion elucidates that the precise measurement of human surface and subcutaneous tissue temperatures using an MWR system is a complex challenge, necessitating an integration of antenna directionality for temperature measurement, radiometer error correction, hardware configuration, and the calibration and precision of a multilayer tissue forward and inversion method. This study delves into the pivotal technologies for non-invasive human tissue temperature monitoring in the microwave frequency range, offering an effective approach for the precise assessment of human epidermal and subcutaneous temperatures, and develops a non-contact microwave protocol for gauging subcutaneous tissue temperature distribution. It is anticipated that mass-produced measurement systems will deliver substantial economic and societal benefits.

## 1. Introduction

According to recent statistics from the World Health Organization, as of 2022, approximately 18.1 million individuals were diagnosed with skin cancer annually [1]. The early detection of skin lesions is crucial for effective treatment and improving patient outcomes, as highlighted by pertinent research. Pathological alterations in skin tissue can be classified into epidermal, dermal, and subcutaneous lesions, depending on their depth [2]. Current conventional diagnostic methods for skin lesions include X-ray photography, CT scans, MRI scans, and ultrasonic imaging, each with distinct advantages and limitations. However, due to the large size and cost of the equipment, the detrimental effects of ionizing radiation, and their limited efficacy in early-stage detection, these techniques are not ideal for widespread early diagnosis. Microwave radiation diagnostic technology emerges as a promising alternative to overcome these issues.

The microwave radiation technique utilizes the fact that all physical objects with temperatures above absolute zero (−273 °C) emit electromagnetic waves, termed radiant heat, in accordance with Planck’s law [3]. This emission results from the random movement of charged particles (electrons, ions, etc.) within the object. The electromagnetic waves emitted by the human body’s internal tissues fall within the decimeter and centimeter bands. Microwave radiometry operates by measuring the body’s inherent thermal radiation energy to gauge the internal temperature of tissues. This non-invasive, harmless approach allows for the monitoring of biological tissue’s thermal activity, facilitating early disease detection as thermodynamic variations precede structural changes in tissues. Importantly, microwave radiometry provides a more detailed analysis of skin temperature compared to infrared thermography, revealing temperature field distribution up to several centimeters deep.

Due to the presence of specific antigens and heightened sensitivity in cancerous cells, viruses and carcinogenic agents can enhance blood circulation, resulting in increased water content within cancerous tissues. Consequently, in the microwave frequency range, the dielectric constant (ε) and electrical conductivity of cancerous tissues exceed those of normal tissues, leading to higher emissivity in cancerous cells. Moreover, cancerous tissues can induce circulatory blockages or infections, which generate heat, raising the local tissue temperature by approximately 1 °C above normal levels [4]. Initially, microwave radiometers were used to identify the approximate locations of tumors through a comparative method. The radiometer captures microwave thermal radiation signals from the cancerous tissue and their symmetrical normal counterparts. The variance in output voltage is utilized to ascertain the existence and approximate location of the cancerous tissue.

A pathological region of skin tissue displays distinct attributes compared to adjacent healthy tissue, including increased temperature, higher water content, and greater dielectric constant and conductivity. These characteristics enhance its sensitivity to microwave radiation, enabling the detection of subtle temperature variations within the affected area [5]. The traditional single-band multi-angle approach for temperature inversion is restricted to measuring the temperature of a specific tissue layer, assuming known temperatures for other layers, which produces a single real temperature parameter. To remove such limitations, it is imperative to explore a nonlinear joint inversion mechanism utilizing the multi-band method under near-field scattering, aiming to accurately determine temperature changes or distributions at various depths within skin tissues. The core principle is that microwave signals across different frequency bands can capture and externalize temperature data from skin tissues at varying depths. Determining the temperature influence of each skin layer involves analyzing the interaction between near-field scattering and system unit variance. By integrating this analysis, the actual temperature values for each layer within the detection area are derived. Given that the temperature of human skin tissue correlates directly with its microcirculation, this relationship can assist physicians in diagnosing skin lesions by examining temperature distributions, comparing them with an existing database, and thus enabling timely intervention and treatment.

In recent years, microwave radiometry has been employed for medical diagnostics in a range of diseases including breast cancer [6], stroke [7], carotid artery atherosclerosis [8,9], activity of brown adipose tissue [10], rheumatoid arthritis [11], joint inflammation [12,13], synovitis [14], varicose veins [15], vesicoureteral reflux [16], urogenital system disorders [17], back pain [18], and diabetic foot [19]. The utility of this method in preclinical research has also been demonstrated in mice [20].

This study presents a systematic examination of multi-band closed-loop forward and inversion modeling for the precise detection of skin tissue temperature. It undertakes extensive theoretical analyses of the critical technologies involved. The research encompasses three primary areas. First, it focuses on enhancing the power transmission efficiency and directional accuracy of the temperature-measuring antenna while elucidating the methods for mitigating non-target radiation interference in the measurement environment. Second, it introduces a highly sensitive correlated radiometer design and a precise calibration approach to minimize the impact of extraneous error factors within the system units. Third, it explores the microwave radiation transmission model in human tissue and develops an efficient temperature inversion algorithm to address the challenge of energy scattering transmission among skin tissues.

## 2. Application of Microwave Radiometry in Biomedical Research

In the 1970s, Enander B. introduced a technique using a Dicke-type microwave radiometer, operating within the frequency band of 0.9 GHz to 1.2 GHz, to detect cancerous tumors by assessing the internal temperature of the human body [21]. Similarly, Barett A.H. and Myers P.C. employed Dicke-type microwave radiometers at center frequencies of 1.3 GHz, 3.3 GHz, 5 GHz, and 10 GHz to measure the human body temperature [22].

This marked the inception of passive microwave applications in the medical field, subsequently encouraging scientists worldwide to conduct related research. Microwave radiometric approaches to measuring the human body temperature can be categorized into contact and non-contact methods. Currently, contact measurements are predominant in the use of microwave radiometers for assessing the human body temperature, with non-contact methods being less common. Table 1 compiles significant studies from the past decade on microwave radiometry in biomedical research, alongside their performance metrics

### 2.1. Contact Thermometry by Microwave Radiometers

Between 1987 and 1995, Xiang X.X. et al. developed the S-band Dicke microwave radiometer diagnostic system [32], characterized by an operating frequency of 2.25–2.65 GHz, with a thermometry sensitivity of up to 0.2 K and a probe receiving a nonlinearly polarized wave with a standing wave ratio (VSWR) of less than 2.0. This system could probe muscular tissue to a depth of 10 cm. Subsequent enhancements from 1989 to 1991 included Li E.Z.’s clinical investigation into detecting nasopharyngeal and esophageal cancers using a thermal radiometer [33,34]. Data were acquired by establishing collection points on either side of the face and neck at corresponding positions, then recording the voltage outputs from two symmetrically placed collection points. A predefined threshold was used; if exceeded, this indicated a potential tumor presence.

From 2000 to 2004, Hand J.W. et al. devised a five-band Dicke radiometer system for monitoring infant brain temperature [35]. Each band featured a multistage low-noise, high-gain amplifier with a 0.4 GHz bandwidth and a 5 s measurement integration time. The system achieved a theoretical luminance temperature resolution of 0, with the temperature measurement precision derived from brightness temperature data through model fitting and Monte Carlo methods to invert the temperature profiles. Each radiometer channel was calibrated to 0.4 K. The 2σ confidence interval for temperature estimations at the head’s center was better than 0.8 K. However, the discrepancy between the inverted and initial temperature values reached up to 1.5 K in the 2D model [36,37].

Between 2008 and 2012, Jacobsen S.K. et al. developed a Dicke-type microwave radiometer operating within a frequency range of 1–4 GHz. This instrument was utilized to assess temperature gradients in human tissue during microwave thermotherapy and to detect bladder urinary reflux in children [38]. The radiometer identified an extremely weak thermal noise signal from the lossy material. To increase the precision of temperature measurements, an active antenna probe was constructed [39]. Tests were conducted on real mannequins and human subjects near the surface, with statistical analysis demonstrating a notable enhancement in the signal-to-noise ratio of the Dicke-type radiometer when employing the active antenna probe versus a standard antenna [40,41,42,43].

From 2013 to 2015, Rodrigues D.B. et al. created a compact microwave radiometer thermometry system for non-invasive monitoring of the average temperature in human tissue up to 5 cm deep [10,44,45,46,47,48]. The radiometer, operating at a center frequency of 1.35 GHz and with a 500 MHz bandwidth, features an EMC logarithmic helical microstrip antenna that emits energy uniaxially with high gain. Evaluations of the radiometric system, through simulation and physical experimental modeling at various clinical measurement sites, demonstrated its ability to accurately monitor temperature increases in human tissues and decreases in brain temperature beneath the scalp and skull. Moreover, it exhibited long-term accuracy and a stability of approximately ±0.4 °C/4.6 h.

Between 2013 and 2017, Scheeler R. et al. explored a wearable microwave radiometer system for measuring subcutaneous temperature fluctuations using a near-field probe [23,49]. Two Dicke-type microwave radiometers, with center frequencies of 1.4 GHz and 2.7 GHz, were employed to simulate human skin. Additionally, dual-frequency and triple-frequency oscillator antennas, with center frequencies of 400 MHz/1.4 GHz and 1.4 GHz/2.7 GHz/4.9 GHz, respectively, were utilized. The human skin model involved two emitters at a center frequency of 1 GHz. Radiometers at 1.4 GHz and 2.7 GHz tested and confirmed the accuracy of the simulated human skin structure. Data inversion was conducted using the least-squares method, and the near-field weighting factor for the antenna was optimized through an optimal estimation approach.

From 2017 to 2018, Haines W. et al. developed a wireless wearable microwave radiometer system for monitoring internal human body temperatures [50]. This system operated within the 1.4–1.427 GHz range, featuring a circular patch antenna probe calibrated to the radiometer receiver using a cold noise source alone. The system’s ability to measure intraoral temperature was demonstrated by recording intrabuccal water temperature, although the initial temperature measurement accuracy was limited. Further development led to a Dicke-type microwave radiometer equipped with a cold/hot noise source. Comparative experiments revealed that the 1.4 GHz Dicke-type microwave radiometer provided more accurate intraoral temperature readings in the human oral cavity than the system calibrated with a thermistor.

From 2019 to 2022, various research teams conducted extensive diagnostic tests on real patients using advanced commercial contact microwave medical devices [12,15,18,51,52]. These tests provided valuable data, enabling physicians to form more comprehensive diagnostic conclusions. However, the current contact diagnostic devices are bulky, and their stability needs to be improved, particularly in terms of their application scope. In medical settings, especially in emergency departments and other critical care environments, non-contact microwave radiometry could expedite the triage process, saving vital treatment time. This technique enables the accurate measurement of body temperature without the need to remove clothing and allows for real-time, non-contact monitoring of body surface temperature at a safe distance, which is particularly beneficial for critically ill patients in ICUs and those with skin tissue damage.

Table 2 details the applications of microwave radiometers in contact measurements, accompanied by a comprehensive analysis of the specific performance indicator values.

### 2.2. Non-Contact Thermometry by Microwave Radiometers

Between 2009 and 2012, Bonds Q. et al. introduced a non-contact technique for measuring human body temperature using a microwave Dicke-type radiometer [53,54]. This approach introduced new challenges for non-contact sensors, especially in antenna design. The team developed a printed dipole antenna for the radiometer, positioned a few centimeters above the tissue model, to record its temperature changes. Although the radiometer successfully tracked the temperature trend, the readings lacked precision. Subsequently, the measurement accuracy was improved by developing an antenna probe with better directivity and capable of uniaxial energy emission, which significantly enhanced the system’s performance over the original microwave Dicke-type radiometer with a printed dipole antenna [55,56].

In 2015, Li Q.X. and Lang L. investigated temperature measurement in cardiac ablation areas using a C-band Dicke-type microwave radiometer. They tested two antenna designs—a double-slit antenna and a monopole bare probe radiometer antenna—to measure the temperature in the cardiac ablation region. The monopole antenna, connected to the radiometer’s input, formed a temperature measurement system. Concurrently, Pi Z.F. explored temperature measurement of a water body using both contact and non-contact approaches [57]. The findings showed a voltage change-to-water temperature change ratio of 19.77 mV/K for contact and 7.36 mV/K for non-contact measurements. He F. employed the multi-angle method to model a five-layer human tissue model, measuring the emitted brightness temperature of a water body with a linear temperature gradient using a C-band Dicke-type radiometer and horn antenna. A calibration scheme was devised using a room-temperature blackbody and one submerged in liquid nitrogen, which allowed for the deduction of water temperatures at varying depths and for determining the total emitted brightness temperature from the water body [24]. The analysis revealed relative errors of 13.9% for the first layer, 8.8% for the second layer, and 4.4% for the third layer.

Table 3 provides a detailed account of the applications of microwave radiometers in non-contact measurements.

In 2017, Park W. et al. devised a microwave radiometer for non-contact and non-invasive human body temperature measurement [25]. This device was calibrated in real-time with two reference noise sources, and a highly directional waveguide horn antenna was utilized to reduce ambient noise interference during the measurements. The team measured water temperature with the radiometer, positioning the antenna approximately 20 cm from the water’s surface. The device demonstrated strong concordance with a standard water thermometer across temperatures ranging from 25.0 °C to 43.1 °C. With linear fitting, measurement inaccuracies were noted as 1.93 K and 0.90 K between 34.5–43.1 °C and 25.0–27.8 °C, respectively, which decreased to 0.62 K and 0.85 K with logarithmic fitting. However, compared to compact planar-type antennas, the designed horn antenna remains bulky for practical applications.

Between 2019 and 2024, various research groups employed custom-built microwave radiation detectors to ascertain the non-contact temperatures of human tissue substitutes. While these efforts somewhat enhanced measurement precision, a clinically applicable medical device has yet to be realized [59,60,61,62,63,64].

## 3. Current Limitations of Microwave Radiometry

Current domestic and international research primarily focuses on the contact temperature measurement of human epidermis and subcutaneous tissue [30,61,62,63,65,66,67,68,69,70,71,72,73,74,75,76,77,78,79,80,81,82,83,84,85]. However, in clinical settings, there is a need to implement non-contact temperature measurements for patients’ epidermis and subcutaneous tissues, along with the capability to continuously monitor within the tissue area. Yet, as of now, non-contact technology for detecting the skin tissue temperature remains undeveloped. Challenges such as antenna pointing accuracy [86,87,88,89,90,91,92,93,94,95], microwave radiometer sensitivity [6,7,16,18,96,97,98,99,100], emissivity and temperature uniformity of calibration sources [101,102,103,104,105,106,107,108,109,110,111,112], as well as the precision and applicability of forward and inverse algorithms [113,114,115,116,117,118,119,120,121] have resulted in the sensitivity and accuracy of microwave temperature measurement systems being above ±0.4 K. This level of performance does not yet meet the standards for medical devices. This paper aims to explore non-contact temperature detection technology for skin and subcutaneous tissues using microwave frequencies. Additionally, it seeks to develop a new multi-band microwave temperature measurement system, leveraging related technologies to fulfill the requirements of current clinical applications and achieve advancements in biological detection technology.

**Figure 1:**
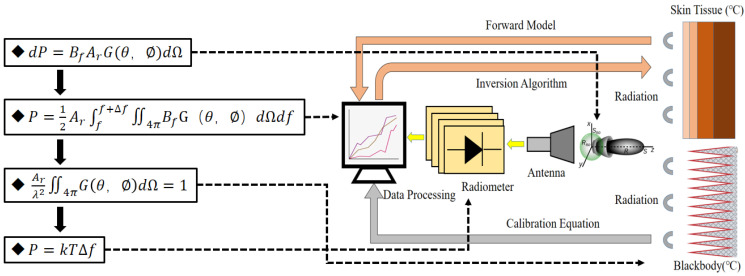
The principle and system composition block diagram of microwave radiation diagnostic technology.

The efficacy of microwave radiometry in practical applications is hindered by its in-adequate temperature measurement accuracy, which does not meet the standards required for clinical use. An examination of the microwave radiometry system and its operating principles, illustrated in Figure 1, reveals key factors contributing to this accuracy limitation. First, the presence of non-target radiation interference within the radiation power received by the temperature-measuring antenna can degrade the performance or hinder the convergence of the temperature inversion algorithm, given the nonlinear radiation interactions among human skin tissues. Thus, addressing the trade-off between the antenna’s small aperture and high directivity is essential. Second, variations in the operational environment of the microwave radiometer can compromise temperature measurement accuracy, indicating that an internal calibration mechanism alone is insufficient. Consequently, there is a need to develop a blackbody calibration source that adjusts for temperature, incorporating both electro-thermal characteristics and the quantification of transfer brightness temperature uncertainty in the calibration process.

## 4. Research Progress and Analysis of Key Technologies

An analysis of current studies reveals that despite extensive investigations into the structure of microwave radiometers and temperature measurement antennas, significant challenges and deficiencies persist. These include inaccuracies in temperature measurement, the antenna’s near-field radiation properties, uncertainties in brightness temperature calibration, the impact of skin tissue temperature on measurement weighting, and a scarcity of research on forward and inversion modeling for assessing the layered temperature distribution in skin tissue. This study introduces a detailed temperature measurement strategy for human skin tissue, employing a multi-band closed-loop approach for forward and inversion modeling. However, urgent attention must be paid to several critical technologies within this strategy. Notably, enhancing the pencil beam radiation characteristics of the temperature measurement antenna through integration with a quantitative model of calibration link uncertainty is crucial. Additionally, defining the correlation between crucial system unit parameters and temperature measurement efficacy is vital. By concentrating on establishing a precise temperature measurement process for human skin tissue through forward and inversion modeling and addressing the effects of various variable parameters and nonlinear scattering on measurement accuracy, this research seeks to develop an early diagnostic system for human skin tissue anomalies that aligns with clinical testing standards. The goal is to advance the theoretical underpinnings of microwave radiometry and lay a robust theoretical foundation for its practical application.

### 4.1. Optimization of Near-Field Radiation Characteristics of Temperature Measurement Antenna and Antenna Structural Parameter Inversion Technology for Pencil-Shaped Beam Distribution

The microwave radiometry system receives extraneous radiation beyond the thermal radiation captured by the antenna’s main lobe. Studies by Duke University, Southeast University, and our group have demonstrated that the system’s sensitivity and accuracy are contingent upon the antenna’s power transmission efficiency and main beam radiation effectiveness. In near-field operations where the major lobe beam width is narrower than 15 degrees, the spatial resolution of the radiometer aligns with the antenna’s aperture size. Thus, developing a highly focused pencil beam antenna is a strategic approach to enhancing the temperature measurement capability for both contact and non-contact human body temperature assessments [46,60,122,123]. Traditional antenna parameter adjustment methods, predominantly reliant on empirical formulas and parameter sweeps, necessitate iterative fine-tuning of antenna structural parameters to balance various performance metrics related to beam distribution. This approach is not only restrictive but also demands considerable time and effort.

Recently, researchers globally have applied neural networks and deep learning to facilitate antenna design [65,66,67,68,124,125,126]. In 2019, Budhu J. et al. at UCLA combined full-wave simulation with particle swarm optimization and physical optics to craft an inhomogeneous medium lens, enhancing the lens antenna’s directivity [124]. In 2020, Wu Q. from Southeast University implemented a Gaussian process regression model to fore-cast the parameters and gain of a microstrip antenna, developing single-output, symmetric, and asymmetric multi-output Gaussian process regression models for various antenna types [125]. The same year, Yuan L. and colleagues at the University of Electronic Science and Technology of China linked reverse and forward neural networks to predict super-surface elements’ structural parameters for specific transmission amplitudes, utilizing transfer function technology despite introducing some errors [126]. They later employed a multi-branch reverse neural network to refine the design, using data classification to manage the electromagnetic problem’s inherent non-uniqueness [65]. For antennas with pencil beam distributions, the need to concurrently consider multiple performance indicators has made multi-objective machine learning approaches particularly relevant. In 2018, Xiao L.Y. and their team at Xiamen University developed three parallel forward neural networks to predict the electro-magnetic parameters of a Fabry–Perot Resonant Cave Antenna, establishing a preliminary mapping relationship using multiple support vector machine models [66]. In 2021, the same group utilized a reverse neural network to estimate the structural parameters of a multimode resonant antenna, although they found that extreme learning machine-based multi-objective evaluation might not always produce optimal outcomes [67]. Also in 2021, Naseri P. from the University of Toronto employed a forward neural network, complemented by a variational auto-encoder, to learn and effectively decode the relationship between the structure, phase, and amplitude of multilayer super-surface elements [68].

In summary, this research presents a strategy for addressing the challenges of high data requirements and the complexity of defining the optimization target in complex electro-magnetic problem-solving. The method employs a reverse neural network as the core element, supplemented by several forward neural networks to provide preliminary knowledge concerning beam distribution. Furthermore, specific equations or parameters are established to streamline the electromagnetic response of the optimization target. This facilitates the implementation of a multi-index optimization algorithm for an all-dielectric lens antenna and the inversion of antenna structural parameters in scenarios involving pencil beam distribution.

### 4.2. Quantification of Uncertainties in Architectural Performance Bottlenecks of Microwave Radiometers and Dual-Electro-Thermal Blackbody Calibration Sources

The accuracy of temperature measurement using the microwave radiation method is influenced by fluctuations and additional errors in each system unit. Since 1974, scholars worldwide have explored the structure of microwave radiometers, identifying performance limitations in both full-power and Dicke-type devices [26,69]. Recently, our team and other researchers have delved into the architecture of radiometers [60,61,62]. While the architecture’s sensitivity is negligible in equilibrium, the correlation radiometer is prone to gain fluctuations during operation, adversely affecting sensitivity. Furthermore, zero drift can also impair measurement accuracy. In 2023, Hu A.Y. and colleagues at Beihang University introduced a coherent radiometer design based on circumferential uniform poly-phase modulation, which mitigates zero drift and minimizes the impact of gain fluctuation on sensitivity [70].

Accurate diagnosis of early skin lesions necessitates precise temperature measurements. However, the existing internal calibration scheme, relying solely on cold/hot noise sources, is insufficient. It is essential to develop a blackbody calibration source with high emissivity and temperature uniformity for external calibration correction. Presently, black-body calibration sources are predominantly of two types: coated cone array and coated cavity. The coated cone array type is favored for its compactness. Investigations by the National Institute of Standards and Technology in the USA and the University of Bern in Switzerland indicate that a calibration source’s brightness temperature depends on the temperature and emissivity performance of its coating. The calibration’s precision is constrained by the absence of established benchmarks and transmission standards for microwave brightness temperature, making it challenging to trace the uncertainty in the radiometer’s brightness temperature measurement [71,72]. Recent efforts by researchers, including our team, have focused on developing quantitative modeling methods for complex radiation targets and near-field receiving antennas [73,74,75,76]. In 2017, Schöder A. and associates at the University of Bern utilized far-field reciprocity in an inverse scattering model to ascertain the local absorption rate and overall reflectivity of a radiator, integrating this with thermal analysis to determine the radiator’s temperature distribution. They introduced a directional radiation brightness temperature model for this purpose [73], indicating a shift toward analyzing overall radiation brightness temperature instead of just emissivity and temperature separately. In 2021, Virone G. et al., from the Italian Institute of Electronic Information and Telecommunications, explored the cone array calibration source’s radiation brightness temperature and its transmission to the antenna through circuit equivalence [74]. They proposed a method to calculate the calibration source’s directional radiation brightness temperature, incorporating the antenna’s far-field pattern and the influence of ambient brightness temperature through specular and diffuse reflection coefficients, leading to the antenna port’s equivalent noise temperature. In 2022, Jin M. and colleagues from Beijing University of Chemical Technology introduced a cone array calibration source design to optimize broadband temperature gradient and absorption performance by adjusting the coating thickness along the cone, achieving a balanced directional radiation brightness temperature with respect to emissivity and temperature gradient [75].

In summary, assessing the impact on the calibration source’s radiation brightness temperature during transmission is challenging due to variables like radiation source distribution, environmental factors, antenna efficiency, and mirror loss. Accordingly, through this review, we seek to support the development of a scattering model for a calibration source, integrating forward and backward modeling theories with the finite element method. The investigation examines the extent to which the calibration source’s electro-thermal characteristics can be considered in terms of overall radiation brightness temperature. Furthermore, this study analyzes how different antenna beams affect the transmission of brightness temperature and determine the uncertainty associated with this transmission. Ultimately, the research is aimed at facilitating the design of highly precise calibration sources and calibration links.

### 4.3. Near-Field Temperature Contribution Weight Function Measurement of Skin Tissue and the Core Difficulty of Temperature Inversion Technology

When employing the microwave radiation method for measuring human tissue temperatures in the near field, the radiation brightness temperature received by the antenna represents the volume-averaged brightness temperature, weighted by the weight function W at the antenna’s entry point within volume V. In the context of measuring human skin tissue’s layered temperatures, it is crucial to recognize that the count of brightness temperature data points exceeds the number of tissue temperature readings, indicating an over-determined set of target parameters. Thus, a multi-band microwave radiometer can be utilized, where the antenna temperature at this juncture can be equated to the matrix representation of the weight function W and the layer temperature vector T. Given the nonlinear nature of energy transmission between skin tissues and the weight function’s dependence on the skin tissues’ dielectric properties and the near-field radiation characteristics of the temperature-measuring antenna, directly measuring the weight function is challenging. Therefore, an inversion algorithm is necessary to resolve the matrix, to derive the layer temperature vector T [23,24,49,77,78]. In 2015, He F. and his team at Huazhong University of Science and Technology employed a Dicke radiometer in the C-band to measure water with a temperature gradient, using the single-frequency-band reading combined with multiple measurement angles as auxiliary parameters to mimic multi-band temperature measurements [24]. In 2019, Qian P.C. and his colleagues at Westmead Hospital in Australia simplified the temperature distribution and weight function inversion process to solving over-determined linear equations, incorporating numerical simulations with an anatomically realistic baby head model to swiftly ascertain the brain’s temperature distribution using data from a multi-band microwave radiometer. This approach also supports error analysis in microwave radiation measurement technology, laying the groundwork for non-invasive body temperature monitoring [78]. Subsequently, research teams from the University of Colorado, Tromso University, and Huazhong University of Science and Technology explored various inversion algorithms like the least-squares method, model fitting, and Monte Carlo methods [23,80], yielding divergent outcomes. Our team introduced a neural network detection model refined by an evolutionary algorithm, though the inversion results have yet to meet expectations [66].

The research highlighted demonstrates that the precision of the weight function calculation is intricately connected to the near-field radiation pattern, dimensions, measure-ment distance, and angle of the temperature-measuring antenna during near-field assessments of skin tissue temperature. Furthermore, variations in human tissue’s dielectric properties can influence the weight function, exacerbating the inaccuracy of the inversion process [81,82,83]. The total radiation power that the antenna receives is a composite of the radiation emanating from the environment, clothing, and skin tissues, introducing numerous varying parameters that significantly constrain the precision of internal body temperature inversion [29,30]. In essence, the fidelity of temperature measurements via the microwave radiation technique is tightly linked to the antenna’s near-field radiation attributes, the calibration link brightness temperature uncertainty, and the temperature contribution weight within the inversion algorithm [84,127,128]. Presently, there is no comprehensive or flawless methodology available, especially considering that the human tissue model and inversion technique necessitate further exploration. With the growing emphasis on the application and theoretical examination of microwave radiometry in the industry, there is an imperative need to expedite research into a layered, precise temperature measurement approach utilizing the multi-band method.

## 5. Implementation Routes of Key Technologies

### 5.1. A Priori Knowledge Neural Network Optimization Model Combining Multi-Node Matching with Q-Value Constraints and Multi-Objective Function Constraints

**Figure 2:**
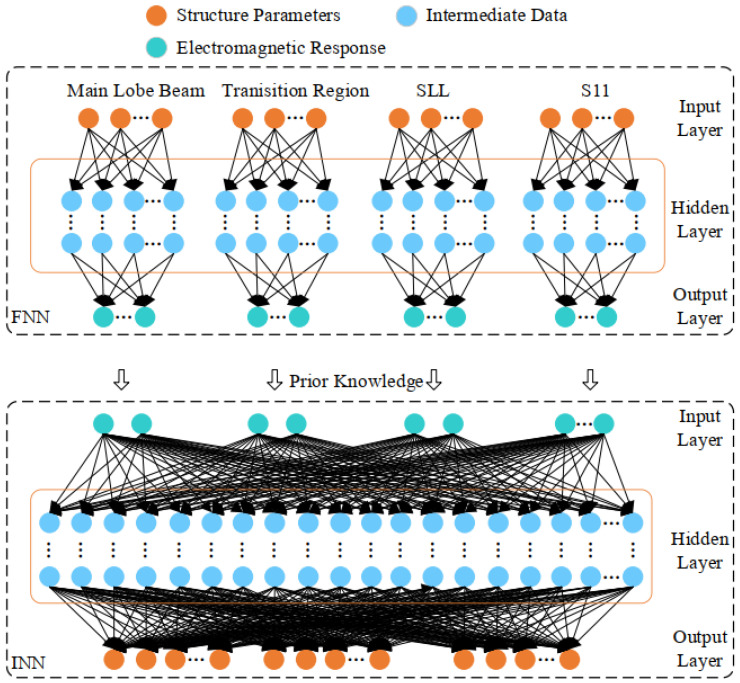
Neural network model based on prior knowledge in this research.

(a) Investigate the factors limiting the voltage standing wave ratio (VSWR) for each structural segment of a temperature-measuring antenna under octave conditions; enhance the antenna’s power transmission efficiency by optimizing VSWR parameters; introduce a Q-constrained multi-branch broadband matching approach utilizing Chebyshev and multi-branch matching theories.
(b) Investigate the drawbacks of manual tuning in antenna optimization; implement an optimization algorithm that integrates swarm intelligence with neural networks; simultaneously target the optimization of the main lobe beam, side-lobe, and transition zone; establish the constraint ranges for various sub-objective functions; adjust weights to enhance the pointing accuracy of the temperature measurement antenna.
(c) Overcome the challenge of excessive data requirements for inverse modeling of the antenna structure; explore a neural network model informed by a priori knowledge; as illustrated in Figure 2, employ multiple sub-forward neural networks (FNNs) for the structural parameter inversion of the antenna, incorporating prior knowledge and multiple indices, culminating in the development of a multi-index optimization system equipped with an ultra-narrow pencil beam temperature-measuring antenna.

### 5.2. Channel Phase Shifting Correction Algorithm and Calibration Link Uncertainty Calibration for Measuring Radiation Brightness Temperature Errors

(a) Develop an error model for the microwave radiometer architecture focusing on key metrics like sensitivity and accuracy; examine how phase, amplitude, offset, and other errors affect radiometer output; devise a periodic phase-shifting error correction algorithm using a uniform polar circle combined with a phase modulation circuit to adjust the detected output data.
(b) Propose a finite element method informed by forward and backward modeling theory to calibrate the scattering model of the calibration source; explore control strategies for the electro-thermal performance of the calibration source, refine its structure, and analyze the impact of the antenna beam on the brightness temperature transmission from the perspective of overall directional radiation temperature; trace the uncertainty in the calibration link and correct the transmission brightness temperature error.

**Figure 3:**
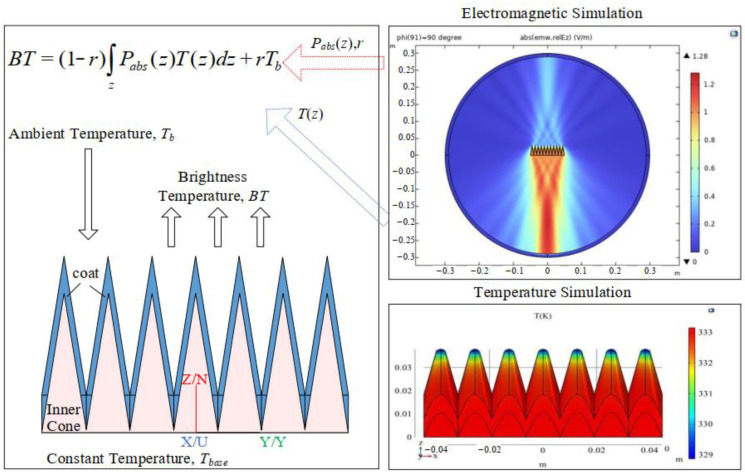
Schematic diagram of the calculation of the brightness temperature for the coating array calibration source’s directional radiation scene.

The radiation brightness temperature for the coated array calibration source is determined using a directional radiation brightness temperature model, predicated on reciprocity in far-field conditions, which enables the calculation of radiation brightness temperature perpendicular to the calibration source’s front direction. Figure 3 illustrates the scenario for calculating the directional radiation brightness temperature for the calibration source.

### 5.3. Incoherent Skin Tissue Radiation Forward Model and Objective Function Constrained Deep Learning Combined Inversion Method

**Figure 4:**
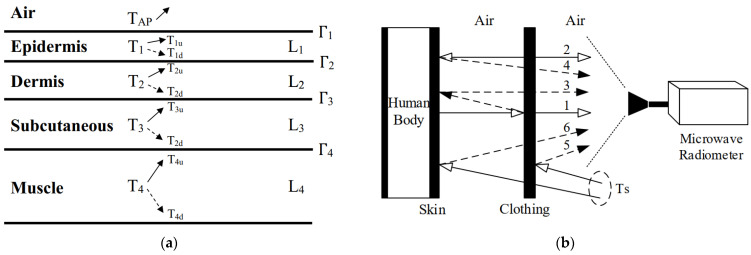
(**a**) Incoherent model of microwave thermal radiation interaction within human skin tissues; (**b**) microwave thermal radiation model for the human body surfaces clad in clothing.

**Figure 5:**
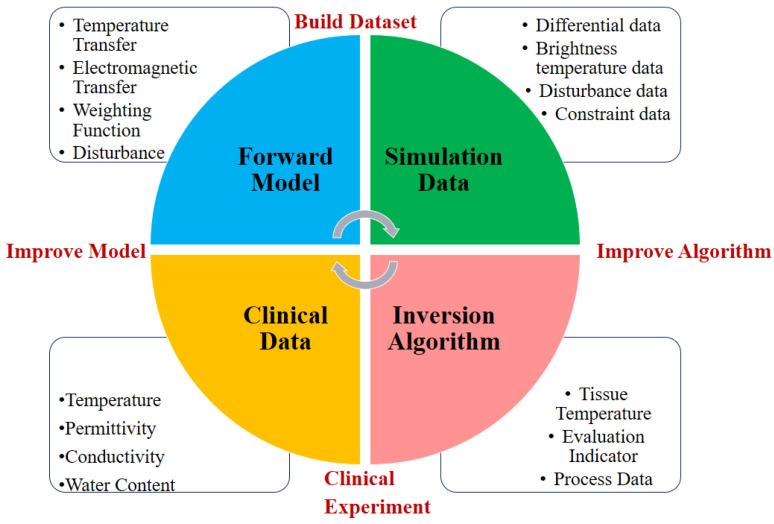
Closed-loop high-precision forward and inversion modeling detection method for human tissue temperature measurement.

(a) Define the relationship between the human skin tissue radiation brightness temperature and the weight function; study the temperature distribution across the human epidermis, dermis, subcutaneous tissue, and muscle layer utilizing C, X, and Ku frequency bands; formulate a mathematical representation of skin tissue heat transfer using an incoherent method; deduce the estimation equation for apparent brightness temperature when the human body’s transmissivity is zero; incorporate scattering effects and establish the forward model for radiation transmission of incoherent skin tissue, as illustrated in Figure 4.
(b) Investigate the factors influencing the accuracy of temperature measurement in near-field conditions; examine the microwave radiation forward model for human skin tissue; determine the constraint range for temperature variations between adjacent skin tissue areas by calculating the contribution weight of each tissue layer’s brightness temperature; establish the objective function for the penalty function correction algorithm.
(c) To enhance the accuracy, generalization, and robustness of the inversion algorithm, introduce a closed-loop high-precision forward and inversion modeling detection method for human tissue temperature measurement, as depicted in Figure 5. Begin by constructing a dataset and defining constraint conditions using the forward model; then, perform tests on human-simulated tissue fluids, skin tissues, and other samples, and collect clinical data to validate the inversion algorithm. A clinical experiment guided by test outcomes and evaluation metrics refines the forward model’s mathematical and physical relationships through comparisons of clinical and simulation data, thereby improving the method’s scientific validity.

## 6. Conclusions and Future Perspectives

Microwave radiometry differs from other prevalent temperature measurement techniques such as thermal imaging, infrared thermometry, and liquid crystal methods, offering significant advantages in diagnosing internal tissue conditions. While these conventional methods generally reflect only the temperature of the epidermal layer, microwave radiometry provides a non-invasive, non-destructive, and harmless means of detecting the thermodynamic changes that precede structural alterations within tissues. According to Planck’s law, the electromagnetic waves radiated by subcutaneous tissues mostly fall within the microwave band, allowing for the detection of temperature changes several centimeters beneath the skin. This capability is particularly advantageous over infrared methods that only gauge surface temperatures. By accessing temperature variations at different depths within the tissues and organs, microwave radiometry can assess internal organ health based on uneven temperature distributions. These temperature profiles are compared against existing databases to form diagnostic conclusions.

This study primarily focused on investigating the key technical challenges associated with non-contact human tissue temperature measurement in the microwave frequency band. Current research, both domestically and internationally, is predominantly oriented towards contact-based measurement of human epidermis temperature. However, for the early diagnosis of skin cancer, it is imperative to measure both epidermal and subcutaneous tissue temperatures, necessitating continuous monitoring of internal tissue regions. Currently, a notable gap exists in the domain of subcutaneous tissue temperature measurement within the domestic context. Factors such as antenna directivity, microwave radiometer sensitivity, calibration source emissivity, temperature uniformity, inversion algorithm accuracy, and applicability prevent the sensitivity and precision of microwave temperature measurement systems from meeting clinical standards. This review embarked on an exhaustive investigation of factors that constrain the accurate measurement of subcutaneous tissue temperature, aiming to support efforts to develop an innovative system for measuring subcutaneous tissue temperature that aligns with clinical accuracy criteria.

At present, there is an urgent need for a new system to meet the accuracy required for human internal tissue temperature measurement technology in clinical applications. The prospects for microwave radiometry include accelerating triage processes in emergency departments, enabling true temperature measurements of clothed individuals, facilitating real-time temperature monitoring at a distance for ICU patients, reducing infection risks for medical staff, ensuring normal patient routines, and monitoring subcutaneous tissue temperatures to assess internal organ health. Additionally, technology’s utility in mass-screening scenarios could significantly aid in effective epidemic control. If fully realized, this technology’s capability to precisely measure core body temperatures could profoundly impact the medical field.

## Figures and Tables

**Table 1:** The last 10 years of representative studies of microwave radiometry in biomedical research.

Year	Author	Architecture of Radiometer	Method of Measurement	Number of Frequency Bands	Accuracy (K)	Sensitivity (K)
2013	Rodrigues D.B. et al. [10]	Total power	Contact	1	0.8	0.4
2014	Scheeler R. et al. [23]	Dicke	Contact	3	0.5	0.2
2015	He F. et al. [24]	Dicke	Non-contact	1	7	2
2017	Park W. et al. [25]	Total power	Non-contact	1	0.85	0.62
2018	Momenroodaki P. et al. [26]	Dicke	Contact	1	0.6	0.4
2021	Vesnin S.G. et al. [27]	Dicke	Contact	1	0.6	0.3
2021	Villa E. et al. [28]	Correlation	Contact	1	0.4	0.15
2022	Streeter R. et al. [29]	Correlation	Contact	1	0.5	0.25
2022	Issac J.P. et al. [30]	Dicke	Contact	2	0.41	0.25
2024	Tian H. et al. [31]	Dicke	Non-contact	1	0.7	0.062

**Table 2:** Selected published studies on contact applications for microwave radiometers.

Reference	Type of Microwave Radiometer	Operation Frequency (GHz)	Performance	Assessed Target
Hand J.W. et al., 2001 [35]	Dicke	1~4	Resolution of 0.07 K Standard error of 0.75 K	Brain of newborn infant
Arunachalam K. et al., 2008 [44]	Digital	3.7~4.2	Resolution of 0.075 K Standard error of 0.217 K	Homogeneous and layered water
Birkelund Y. et al., 2011 [38]	Dicke	3~4	Standard error of 0.8 K Detection depth of 8 mm	Urine inside a pediatric bladder
Rodrigues D.B. et al., 2013 [10]	Total power	1.5~2.2	Resolution of 0.4 K Detection depth of 12 mm	Multilayer 3D computational model of skin, subcutaneous fat, muscle, and a BAT region located between fat and muscle
Stauffer P.R. et al., 2014 [47]	Total power	1.1~1.6	Maximum error of 0.4 K Correlation (r = 0.9979)	Head model with separate brain and scalp regions
Popovic Z. et al., 2014 [49]	Dicke	1.4, 2.7	Resolution of 0.2 K Minimum error of 0.5 K	Skin, fat, and muscle
Haines W. et al., 2017 [50]	Total power	1.4~1.427	Maximum error of 0.6 K Detection depth of 8 mm	Phantoms of muscle, fat, and skin
Momenroodaki P. et al., 2018 [26]	Dicke	1.4~1.427	Resolution of 0.4 K Minimum error of 0.6 K	Human cheek and mouth
Ravi V.M. et al., 2019 [51]	Total power	1.0~1.6	Resolution of 0.25 K Standard error of 0.4 K	Knee joints
Laskari K. et al., 2020 [12]	RTM-01-RES	1.14, 3.8	Standard error of 0.4 K Detection depth of 7 cm	Small and large joints (hand/arm, foot/leg, wrist, elbow, knee, ankle); sacroiliac joints
Tarakanov A.V. et al., 2021 [13]	MWR-2020	3.4~4.2	Accuracy of 0.2 K Detection depth of 7 cm	Knee
Tarakanov A.V. et al., 2021 [18]	MWR-2020	3.4~4.2	Accuracy of 0.2 K Detection depth of 7 cm	Lumbar spine
Tarakanov A.V. et al., 2022 [52]	MWR-2020	3.4~4.2	Accuracy of 0.2 K Detection depth of 7 cm	Lumbar spine
Levshinskii V. et al., 2022 [15]	MWR-2020	3.4~4.2	Accuracy of 0.2 K Detection depth of 7 cm	Lower extremities and their models

**Table 3:** Selected published studies on non-contact applications for microwave radiometers.

Reference	Type of Microwave Radiometer	Type of Antenna	Central Frequency (GHz)	Bandwidth (GHz)	Performance	Assessed Target
Stephan K.D. et al., 2007 [58]	Total power	Microstrip array antenna	12.5	0.47	Accuracy of 4 K Detection depth of 2 mm	Hamburger patty
Bonds Q. et al., 2009 [53]	Total power	Printed dipole antenna	1.4	0.4	Accuracy of 4 K Detection depth of 5 cm	Muscle tissue phantom
Bonds Q. et al., 2009 [55]	Total power	Cavity-backed slot antenna (CBSA)	1.4	0.4	Accuracy of 1.5 K Detection depth of 2 cm	Skin tissue phantom
Pi Z.F. 2015 [57]	Dicke	Monopole bare probe cap antenna	4.15	4	Resolution of 0.6 K Accuracy of 0.8 K	Water
He F. et al., 2015 [24]	Dicke	Horn antenna	4	1	Resolution of 2 K Accuracy of 7 K	Water of different depths
Park W. et al., 2017 [25]	Total power	Horn antenna	3	0.23	Resolution of 0.62 K Accuracy of 0.85 K	Water
Ravi V.M. et al., 2018 [59]	Dicke	SIW slot antenna	1.3	0.2	Resolution of 0.6 K Detection depth of 45 mm	Tissue phantom
Sun G.M. et al., 2021 [60]	Correlation	Horn antenna	5	2	Resolution of 0.4 K Maximum error of 0.5 K	Water
Sun G.M. et al., 2021 [61]	Correlation	Horn antenna	14	4	Sensitivity of 0.047 K/mV Detection of 215 mV/dBm	Water
Liu J. et al., 2023 [62]	Correlation	Horn antenna	14	4	Average error of 0.034 K Detection of 299 mV/dBm	Palm
Tian H. et al., 2023 [63]	Dicke	Horn antenna	15	6	Resolution of 0.08 K Maximum error 0.6 K	Water, swine skin tissue
Tian H. et al., 2024 [31]	Dicke	Horn antenna	15	6	Resolution of 0.062 K Maximum error 0.7 K	Water sheltered by 5-layer cotton cloth
Liu J. et al., 2024 [64]	Correlation	Horn antenna	10, 14, 16	4	Mean absolute error of 0.5921 K Root mean squared error of 0.6387 K	Swine skin tissue

## Data Availability

Data sharing is not applicable to this article.
